# Evaluation of Nucleic Acid Preservation Cards for West Nile Virus Testing in Dead Birds

**DOI:** 10.1371/journal.pone.0157555

**Published:** 2016-06-24

**Authors:** Leslie Foss, William K. Reisen, Ying Fang, Vicki Kramer, Kerry Padgett

**Affiliations:** 1 California Department of Public Health, Vector-Borne Disease Section, 850 Marina Bay Parkway, Richmond, California, United States of America; 2 Davis Arbovirus Research and Training Laboratory, Department of Pathology, Microbiology and Immunology, School of Veterinary Medicine, 3331 VetMed3A, University of California Davis, Davis, California, United States of America; 3 California Department of Public Health, Vector-Borne Disease Section, 1616 Capitol Avenue, MS-7307, P.O. Box 997377, Sacramento, California, United States of America; U.S. Geological Survey, UNITED STATES

## Abstract

The California West Nile virus (WNV) Dead Bird Surveillance Program (DBSP) is an important component of WNV surveillance in the state. We evaluated FTA™ and RNASound™ cards as an alternative method for sampling dead birds for WNV molecular testing as these cards allow for more cost effective, rapid, and safer diagnostic sampling than the shipment of bird carcasses. To evaluate accuracy of results among avian sampling regimes, Reverse-Transcription Polymerase Chain Reaction (RT-PCR) results from FTA™ and RNASound™ cards were compared with results from kidney tissue, brain tissue, or oral swabs in lysis buffer in 2012–2013. In addition, RT-PCR results were compared with results from oral swabs tested by rapid antigen tests (RAMP™ and VecTOR™). While test results from the cards were not as sensitive as kidney tissue testing, they were more likely to provide accurate results than rapid antigen tests, and detected WNV in corvids as well as in other passerines, raptors, and waterfowl. Overall, WNV RT-PCR cycle threshold (Ct) scores from the cards were higher than those from tissue testing, but both card products displayed high sensitivity and specificity. American Crow samples provided the highest sensitivity. The cards also proved to be easier and more convenient vehicles for collecting and shipping samples, and in 2014 our program launched use of RNASound™ cards in the DBSP. Both FTA™ and RNASound™ products displayed 96% agreement with tissue results and are an adequate alternative sampling method for WNV dead bird testing.

## Introduction

In 1999, West Nile virus (WNV, *Flaviviridae*, *Flavivirus*), was introduced into the US in New York City [1]. The virus has since become endemic in North America, maintained by enzootic transmission between birds and mosquitoes. As an arbovirus (a virus spread by an arthropod), WNV also infects humans and other mammals as incidental hosts after a bite from an infected mosquito. Although many bird species are susceptible to illness and death from WNV, crows and other corvid species (family Corvidae) are highly susceptible and large scale die-offs of these birds have been a strong indicator of outbreaks of human disease [1, 2]. Monitoring spatial and temporal patterns of dead bird clusters has provided an excellent tool for tracking WNV activity [3, 4, 5].

Since 2000, the California Department of Public Health (CDPH) has tested dead wild birds as part of the WNV Dead Bird Surveillance Program (DBSP) in collaboration with university researchers and a multitude of local agencies, including vector control districts and environmental and public health departments [6, 7]. In addition to testing dead birds, the comprehensive WNV surveillance program tests mosquito samples, sentinel chickens, and incorporates human case data, with periodic monitoring of horses and tree squirrels. This surveillance data is used by local agencies to locate and control WNV-positive mosquito populations and alert their citizens about potential human WNV infection risk. The California public can report dead birds via a Dead Bird Hotline (877-WNV-BIRD) and website (www.westnile.ca.gov). CDPH hotline personnel screen reports for suitable specimens (birds dead < 24 hr, non-trauma deaths), dispense safe carcass handling instructions to the public, and dispatch directions to local agencies for dead bird pick-up.

Dead bird carcasses have been tested by a variety of methods. From the initiation of the DBSP until late in 2013, the majority of collected dead birds were shipped to the California Animal Health and Food Safety Laboratory (CAHFS) for necropsy, where kidney tissue and oral swab specimens were collected, preserved in lysis buffer, and tested for WNV ribonucleic acid (RNA) at the University of California, Davis Arbovirus Research and Training Laboratory (DART). Although testing necropsied samples is considered the most accurate method, it required time and temperature-sensitive steps (e.g., maintaining the carcass at a cold temperature; packing and shipping within 24-48h of collection) and was labor and cost intensive [8]. The carcass and tissue samples also required handling according to biohazardous material safety regulations during and after necropsy. Rapid antigen tests (RAMP™ and VecTOR™) have been utilized by some vector control agencies for on-site dead bird testing and immediate results [8]; however, these detection assays are less sensitive than RT-PCR [9].

While corvids collectively comprise the majority of WNV-positive specimens submitted for testing in the state each year, non-corvid species such as the House Finch *(Haemorhous mexicanus)*, House Sparrow *(Passer domesticus)*, Northern Mockingbird *(Mimus polyglottos)*, and Red-tailed Hawk *(Buteo lineatus)* are often reported dead by the public and test WNV- positive in California [10]. Oral swab samples have been found to be a good surrogate to necropsy of kidney tissue but have been validated by RT-PCR only for corvids [8]. Prior to this study, it was unclear whether non-corvid bird species have sufficient virus in their oral cavity to allow detection of WNV by molecular methods using oral samples.

To improve efficiency, enhance safety and reduce costs, we sought an alternative method to test dead birds for WNV. The new method needed to be free of biohazardous shipment requirements (i.e., no carcasses or liquid samples) and temperature constraints (i.e., not require ice packs, dry ice, or uninterrupted cold storage). Filter paper is a common sample substrate used in diagnostics for a variety of pathogens. We found that two filter paper nucleic acid preservation card products, FTA™ cards (Flinder’s Technology Associate; Whatman, Florham Park, NJ) and RNASound™ cards (FortiusBio, LLC; San Diego, CA), fulfilled these requirements and allowed RT-PCR testing for viral RNA. FTA™ cards have been widely used to preserve viruses for later analyses (see results in [11, 12, 13, 14] and Rocky Baker, pers. communication). Our study evaluated the efficacy of FTA™ and RNASound™ cards as a sensitive and specific means of detecting WNV in dead birds.

## Materials and Methods

### Dead Birds

Permission was granted for members of the public and partnering local agencies to salvage dead birds, lagomorphs, and rodents for WNV detection in California, through a memorandum of understanding between CDPH and the California Department of Fish and Wildlife (CDFW). Threatened, endangered, or fully protected species (state or federal) are not included in this agreement and were reported and/or surrendered to CDFW upon identification. If dead birds were located on private properties, verbal or written permission was obtained to access properties for carcass collection. Animal ethics protocols did not apply in this study since no live animals were used.

### FTA MicroIndicator™ cards

FTA MicroIndicator™ cards have a pink-colored circular sampling area that turns white when wet to indicate sufficient sample on the card [15]. Samples are swabbed or dotted onto the card and allowed to dry. Since the sample’s DNA or RNA is inactivated and preserved, the cards can be shipped or stored without biohazardous risk.

### RNASound™ Cards

FortiusBio, LLC (San Diego, CA) RNASound™ filter paper cards stabilize and allow detection from RNA samples kept at room temperature for up to a week. RNASound ReadyPunched™ cards are similar to FTA MicroIndicator™ cards (hereafter referred to as RNASound™ cards and FTA™ cards, respectively) in appearance: they accommodate one sample area per card in a printed ring and open match-book style. However, RNASound™ cards feature two 5-mm perforated discs that take up to 10uL each of sample. The discs may be detached with a pipette tip, eliminating the need for punching and sterilizing of instruments between samples thereby reducing potential cross-contamination. The cards also integrate sampling and extraction; nucleic acids are eluted in water and no further RNA purification step is needed [16].

### Trial I: June—August 2012

#### FTA™ Cards

A subset of dead birds collected through the WNV DBSP had an additional swab retained on the FTA™ nucleic acid based preservation cards. Following routine dead bird testing, dead birds in suitable condition were shipped to CAHFS with a cold pack during June, July, and August (typically peak WNV season) 2012. Oral swab samples from American Crows (*Corvus brachyrhynchos*), Western Scrub-jays (*Aphelocoma californica*), and Yellow-billed Magpies (*Pica nuttalli*), were taken in a biological safety cabinet before routine necropsies at CAHFS. A sterile nylon-tipped swab was used to swab the bird’s oropharyngeal cavity, and the sample was pressed and rolled onto an FTA™ card inside the printed ring. The cards were allowed to dry in the safety cabinet for 1 hr before the flap was closed. Cards were then delivered to the DART laboratory, where three 4-mm round discs were punched using disposable biopsy punches (Uni-Punch™, Premier Medical Products, Plymouth Meeting, PA). The discs were eluted overnight in 1 mL ABI Magmax lysis binding solution concentrate (AM8500, Applied Biosystems, Grand Island, NY), and analyzed by real-time reverse transcription-polymerase chain reaction (RT-PCR) using previously published primers [17]. In 2013, instead of using disposable punches, we used a 3.5mm stainless steel rodent ear punch (Kent Scientific, Torrington, CT) to punch four discs from the FTA™ cards. The punch was decontaminated with alcohol and a flame and cooled on dry ice before punching each card.

After oral swabs were taken for FTA™ filter card sampling, an additional oral swab sample was taken and swirled in a vial of lysis buffer, the stem broken off, and cap closed. Dead birds were then necropsied to obtain a ‘lentil-sized snip’ of kidney tissue. Both kidney tissue and oral swab samples stored in lysis buffer were analyzed at DART using RNA extraction and qRT-PCR procedures similar to those described for mosquito pools [18]. Birds with cycle threshold (Ct) scores < 30 were considered WNV positive; Ct scores > 30 and < 40 were confirmed using the original primers and those from the NS1 region [19]. The test result category “chronic infection” was developed to discern between recent or acute infections in birds containing a high virus load (Ct < 30), and birds with a low virus load (Ct > 30 and < 40) that probably were infected in the past [20, 21]. Based on our standard curves, Ct scores ≥ 30 have a WNV titer of ≤ 1 plaque forming unit of virus per mL. This category was in place from 2010 to 2013 when kidney samples were tested. We compared FTA™ and oral swab Ct scores using positive ≤ 40 and negative > 40.

### Trial II: July—August 2013

#### FTA™ and RNASound™ cards

Trial II expanded upon Trial I in scope of participation, avian species sampled, and card products tested. Three vector control agencies (Greater Los Angeles County Vector Control District (VCD), Sacramento-Yolo Mosquito and Vector Control District (MVCD), and Placer MVCD) sampled corvids, other passerine species, and raptors by collecting oral swabs on FTA™ and RNASound™ cards according to the methods described above. The species of dead birds sampled was subject to those found dead and reported by the public. Cards were shipped with the dead birds to CAHFS, where the birds were necropsied and their kidney tissue analyzed as described above. RNASound™ card sampling was identical to that of FTA™ cards, except the drying time before closing the card flap was 2 hr. Filter cards were tested in parallel with kidney tissue by RT-PCR in all birds, and in parallel with RT-PCR analyses on oral swabs in lysis buffer taken from American Crows. **Brain Tissue Analysis:** Brain tissue, obtained via needle aspiration, is an alternative sample type that may provide comparative results to kidney tissue for WNV testing (Dr. Paula Macedo, pers. communication). A limited number of agencies utilize brain tissue to sample bird carcasses. Brain tissue RT-PCR Ct scores were also compared to those from FTA™ and RNASound™ cards, as well as DART tissue RT-PCR.

### Statistics

Means and standard errors of Ct scores among all tests were verified for similarities, and normal distributions of data were verified prior to analyses. Student’s two-tailed paired T-tests (Microsoft Excel 2010, Redmond, WA) were used to compare scores between FTA™ and RNASound™ filter cards, kidney tissue, oral swab, and agency testing (either brain tissue or additional oral swab RT-PCR).

## Results

### Trial I

Between June 27 and August 14, 2012, FTA™ cards with oral swab samples as well as kidney samples and oral swab samples collected in lysis buffer from 25 American Crows, and FTA™ card and kidney samples from 20 Western Scrub-jays and 10 Yellow-billed Magpies were tested. Based on the criterion that Ct ≤ 40 is positive, overall agreement between the two test methods was 82% (45/55) (Table 1). Six corvids with chronic kidney Ct scores (30–40) had negative FTA™ card results. Four birds with negative kidney samples had positive FTA™ cards and these were classified as false positives (Table 1). Overall FTA™ card sensitivity was 25/31 = 0.81 and specificity was 20/24 = 0.83. Also of note, the positive rate of FTA™ cards (29/55 = 53%) is similar to the positive rate of kidney samples (31/55 = 56%).

**Table 1:** Summary of West Nile virus test results for dead corvids: oral samples on FTA™ cards, and parallel kidney tissue (RT-PCR). Trial I, June-August 2012, California. AMCR = American Crow; WESJ = Western Scrub-jay; YBMA = Yellow-billed Magpie.

FTA Card	Kidney	Number	Assessment	Species
Negative	Negative	20	True Negative	14 AMCR, 6 WESJ
Positive	Positive	25	True Positive	9 AMCR, 9 WESJ, 7 YBMA
Positive	Negative	4	False Positive: FTA	2 WESJ, 2 YBMA
Negative	Chronic*	6	FTA > kidney**	2 AMCR, 3 WESJ, 1 YBMA
Total		55		

*Chronic = Ct score from kidney samples > 30; considered positive.

**FTA > kidney = Ct scores from FTA™ cards were higher than Ct scores from kidney samples.

#### Trial I: American Crows

In American Crows alone, sensitivity and specificity of FTA™ cards were higher (15/15 = 1.00 and 9/10 = 0.90, respectively) based on kidney tissue results, and 100% accurate based on oral swab RT-PCR results (both sensitivity and specificity were 1.00). Ct scores were available for n = 9 WNV RNA positive American Crows (Ct scores were not reported for negative results). Oral swabs preserved in lysis buffer scored an average of 5.5 Ct higher than those from kidney tissue (*P* < 0.05) (Table 2). However, oral swabs in lysis buffer results were similar to FTA™ cards with preserved oral swabs (average difference of 1.6, with FTA™ card scores higher in eight of the nine crows; *P* = 0.07) (Table 2). Finally, FTA™ cards with preserved oral swabs had Ct scores that were significantly higher than kidney tissue scores from the same birds (average of 7.0 Ct higher; *P* < 0.05) (Table 2).

**Table 2:** Average cycle threshold (Ct) score differences, standard errors, and *P*-values for T-tests conducted on RT-PCR results from West Nile virus testing of dead corvids. Comparisons between: oral samples on FTA™ cards, kidney tissue, and oral swab (American crows) analyses. Trial I, June-August 2012, California. Underlined test methods indicate the higher (therefore less viral RNA detected) scores in each pair. Ct scores were available for n = 24 of the 55 birds tested.

	T-Tests: Average difference in Ct scores with standard errors and *P*-values			
Species	FTA vs. Kidney	FTA vs. Oral Swab	Oral Swab vs. Kidney	n
American Crow	7.0 (0.72) *P* < 0.05	1.6 (0.44) *P* = 0.07	5.5 (0.82) *P* < 0.05	9
Western Scrub-jay	8.1 (0.54) *P* < 0.05	n/t	n/t	9
Yellow-billed Magpie	9.3 (0.89) *P* < 0.05	n/t	n/t	6
Total				24

#### Trial I: Western Scrub-jays and Yellow-billed Magpies

Since oral swabs in lysis buffer samples are less accurate for other corvid species [8], kidney tissue results were compared to FTA™ card results for Western Scrub-jays and Yellow-billed Magpies. Ct scores from FTA™ cards averaged 8.1 Ct higher than kidney tissue scores in Western Scrub-jays (*P* < 0.05; n = 9) (Table 2). The difference was even greater in Yellow-billed Magpies (FTA™ cards were 9.3 Ct higher; *P* < 0.05; n = 6) (Table 2). FTA™ card false positives occurred in two Western Scrub-jays and two Yellow-billed Magpies (FTA™ cards positive; kidney tissue negative) (Table 1). Both FTA™ card and tissue samples from three of these birds were retested with the same results.

### Trial II Results

During the 2013 WNV season, the species of birds included in Trial II was expanded to include other passerine and raptor species (n = 41) (Table 3). One American Crow and two House Finches were tested only by comparative tissue types. **Agency testing:** Greater Los Angeles VCD did not conduct in-house testing (n = 9; Table 3). Sacramento-Yolo MVCD tested oral swabs from American Crows (n = 17) and brain tissue samples from other species by RT-PCR (n = 15) (Table 3). Placer MVCD tested both oral swab and brain tissue samples by RT-PCR (n = 8) (Table 3).

**Table 3:** Counts of dead birds sampled by three vector control agencies for FTA™ and/or RNASound™ card testing for West Nile virus, which were compared to parallel tissue testing (RT-PCR) at the UC Davis Arbovirus Research and Training Laboratory. Trial II, July-August 2013, California. Additional tissue testing (RT-PCR) by agencies is in parentheses.

	Agency		
Species (Corvids)	Greater Los Angeles VCD*	Sacramento-Yolo MVCD**	Placer MVCD**
American Crow *(Corvus brachyrynchos)*	5	17 (Oral Swab)	
Common Raven *(Corvus corax)*		1 (Brain)	
Western Scrub-jay *(Aphelocoma californica)*		4 (Brain)	1(Oral Swab, Brain)
Yellow-billed Magpie *(Pica nuttalli)*		2 (Brain)	
**Species (Non-corvids)**			
Acorn Woodpecker *(Melanerpes formicivorus)*		1 (Brain)	
American Robin *(Turdus migratorius)*		1 (Brain)	1 (Oral Swab, Brain)
Cooper's Hawk *(Accipiter cooperii)*	1		
House Finch *(Haemorhous mexicanus)*	2	3 (Brain)	1 (Oral Swab, Brain)
House Sparrow *(Passer domesticus)*		1 (Brain)	
Northern Mockingbird *(Mimus polyglottos)*		2 (Brain)	
Orchard Oriole *(Icterus spurius)*	1		
Red-winged Blackbird *(Agelaius phoeniceus)*			1 (Oral Swab, Brain)
Unknown Finch Species			1 (Oral Swab, Brain)
Unknown Species			1 (Oral Swab, Brain)
White-crowned Sparrow *(Zonotrichia leucophrys)*			2 (Oral Swab, Brain)
Totals	9	32	8

*Vector Control District

**Mosquito and Vector Control District.

#### Trial II: American Crows

Both oral swab and kidney tissue RT-PCR tests were conducted on American Crows. The two test methods agreed in 18 birds: 2 were WNV negative (Ct > 40), and 16 were WNV positive (Ct ≤ 30). Oral swab Ct scores in positive crows averaged 4.0 Ct higher than kidney tissue (n = 16; *P* < 0.05) (Table 4). FTA™ card scores were an average of 2.3 Ct higher than scores of oral swabs in lysis buffer, but FTA™ card scores were higher than those of oral swabs in six crows, and were lower than those of oral swabs in another six crows (*P* = 0.05; n = 12) (Table 4). FTA™ cards averaged 5.9 Ct higher than kidney Ct scores (*P* < 0.05; n = 11) (Table 4); FTA™ card scores were higher than kidney scores in all but one of 11 American Crows.

**Table 4:** Average cycle threshold (Ct) score differences, standard errors, and *P*-values for T-tests conducted on RT-PCR results for: oral samples on FTA™ cards, kidney tissue, and oral swab (American Crows) analyses from corvid species. Trial II, July-August 2013, California. Bolded test methods indicate the higher (therefore less viral RNA detected) scores in each pair. Ct scores were available for n = 41 of the 58 birds tested. (AMCR = American Crow; WESJ = Western Scrub-jay; YBMA = Yellow-billed Magpie).

	Trial II: Average difference in Ct scores with standard error, *P-*values, and n		
Species	FTA vs. Kidney	FTA vs. Oral Swab	Kidney vs. Oral Swab
AMCR	5.9 (1.0) *P* < 0.05; n = 11	2.3 (0.51) *P* = 0.05; n = 12	4.0 (0.62) *P* < 0.05; n = 16
WESJ	8.0 (1.7) *P* = 0.13; n = 2	n/t	n/t
YBMA	3.1; n = 1	n/t	n/t

#### Trial II: Multiple Species and Filter Card Comparisons

Overall, FTA™ cards and/or RNASound™ cards correctly identified 24 WNV positive and 19 WNV negative birds representing 16 known species (Table 5). Overall agreement between the filter card products results and tissue testing results was 96% (43/45). Not every test was performed on every bird, but most dead birds were tested by at least two of the five test types. We compared positive Ct scores among test types from the same dead birds (Table 6). Both FTA™ card and RNASound™ card Ct scores were significantly higher than kidney tissue scores (*P* < 0.05 for both comparisons; n = 15 FTA™ cards and n = 22 RNASound™ cards), with overall mean differences between the two test types of 6.0 to 6.6 Ct, or about 2 orders of magnitude WNV RNA concentration (each Ct increase in 3 indicates one order of magnitude, or 10-fold decrease, in viral RNA). As previously stated, Ct scores from FTA™ cards were significantly different from oral swab scores in American Crows (*P* = 0.05; n = 12) (Tables 4 and 6), and there were also differences between those of RNASound™ cards and oral swabs (*P* < 0.05; n = 17) (Table 6). Scores between FTA™ and RNASound™ cards were not significantly different from one another (*P* = 0.25; n = 16) (Table 6), nor were those tests conducted by the local agencies different from DART laboratory scores (kidney tissue tested by DART vs. brain tissue tested at Sacramento-Yolo MVCD: *P* = 0.38; n = 9; no brain tissue vs. kidney comparisons from Placer MVCD were available). There was also no significant difference between oral swab samples tested by DART vs. oral swab samples tested by local agencies (*P* = 0.24; n = 13) (Table 6).

**Table 5:** Summary of West Nile virus test results for multiple bird species: oral samples on FTA™ cards and/or RNASound™ cards compared to one or more results from kidney tissue, oral swab, or brain tissue analyses (RT-PCR). Trial II, July-August 2013, California.

FTA™ and/or RNASound Card™	Tissue(s)	Number	Assessment	Species
				American Crow (16)
				Western Scrub-jay (3)
Positive	Positive	24	True Positive (FTA™ and	Yellow-billed Magpie (2)
			RNASound™)	American Robin (1)
				House Finch (1)
				Unknown spp. (1)
				American Crow (4)
				Western Scrub-jay (2)
				Acorn Woodpecker (1)
				American Robin (1)
				Cooper’s Hawk (1)
Negative	Negative	19	True Negative (FTA™ and	House Finch (3)
			RNASound™)	House Sparrow (1)
				Northern Mockingbird (1)
				Orchard Oriole (1)
				Red-winged Blackbird (1)
				White-crowned Sparrow (2)
				Unknown Finch (1)
Total		43		

**Table 6:** Average cycle threshold (Ct) score differences, standard errors and *P*-values for T-tests conducted on RT-PCR results for several bird species. Comparisons between: oral swab samples on FTA™ or RNASound™ cards, kidney tissue, oral swabs (American crows) and local agency (oral swab or brain) testing (all tested by RT-PCR). Trial II, July-August, 2013, California. Bolded test methods indicate the higher (therefore lower viral RNA detected) scores in each pair. All analyses were conducted by the UC Davis Arbovirus Research and Training Laboratory, except where local agency testing is indicated. N = 48 birds were tested by the various test methods.

Comparison	n	Ave. Ct score difference |x1-x2| / n	Standard Error	*P*
**FTA** vs. Kidney	15	6.0	1.0	< 0.05
**FTA** vs. Oral Swab	12	2.3	0.51	= 0.05
**RNASound** vs. Kidney	22	6.0	0.78	< 0.05
**RNASound** vs. Oral Swab	17	2.0	0.40	< 0.05
FTA vs. RNASound	16	2.6	0.59	0.25
Kidney vs. Brain (agency)	9	5.4	1.21	0.38
Oral Swab vs. Oral Swab (agency)	13	1.9	0.62	0.24

#### Trial II: Discrepancies or Notable Differences

Although there was not a significant difference between results from brain tissue and kidney tissue, inconsistencies in three dead birds were notable. A Western Scrub-jay’s brain tissue Ct score was 21.65, whereas the kidney tissue score was much lower at 13.2 (for a difference of 8.45 Ct). Scores from both FTA™ and RNASound™ cards tested from this bird agreed more closely with the (higher) brain tissue score (Ct range ~19–22). Conversely, the Common Raven’s brain tissue Ct score was 21.88 whereas the kidney score was much higher at 34 (for a 12+ difference). The other two tests conducted on this carcass (DART oral swab and RNASound™ card) agreed more closely with the (higher) kidney tissue score (Ct > 34). Another discrepancy in results occurred in a Northern Mockingbird: the RNASound™ card Ct score was 29, whereas both the kidney and brain tissue were negative for WNV. **Sensitivity and Specificity:** As in Trial I, sensitivity and specificity for Trial II results were calculated based on positive/negative criterion of Ct ≤ 40 = WNV positive. FTA™ cards had a sensitivity of 16/16 = 1.0 and specificity of 4/5 = 0.80 (n = 21), whereas RNASound™ cards had a sensitivity of 28/29 = 0.97 and a specificity of 19/20 = 0.95 (n = 49).

#### Operational Results during 2014

In the 2014 WNV season, 24 agencies participated in the newly implemented RNASound™ card sampling and testing protocol, where 694 birds were tested for WNV RNA; 323 of which tested positive (47%). Positive birds included 23 species: corvids: American Crow (183/307 positive, or 60%), Common Raven (7/20; 35%), Steller’s Jay (5/11; 45%), Western Scrub-jay (73/123; 59%), and Yellow-billed Magpie (2/3; 67%); other species: House Sparrow (9/23; 39%), House Finch (6/14; 43%), and Northern Mockingbird (3/8; 38%), American Robin *(Turdus migratorius)* (3/12; 25%), and Western Bluebird *(Sialia mexicana)* (3/4; 75%), as well as other passerines, raptors, and waterfowl. RNASound™ cards tested negative from over 50 species of dead birds, also including corvids, other passerines, raptors and waterfowl. Additionally, some local agencies conducted in-house RT-PCR testing of various tissues from dead birds and detected WNV in 819 of out of 1,134 dead birds (72%). Overall West Nile virus prevalence in dead birds was 60% in 2014 which was a record high in the California DBSP.

## Discussion

FTA™ cards with preserved RNA from dead bird oral swabs exhibited high sensitivity and specificity in detecting WNV RNA as also reported in [22]. FTA™ cards and RNASound™ cards also performed with high sensitivity and specificity in Trial II. Our results were consistent with previous testing at Oregon State University Veterinary Diagnostic Laboratory, which assessed the efficacy of using FTA™ cards for WNV dead corvid testing and found high accuracy between FTA™ cards and brain tissue results (Rocky Baker, pers. communication).

While FTA™ and RNASound™ cards with oral swab samples generally had lower sensitivity than kidney or brain tissues, as shown by the higher Ct scores; the number of false negative samples was relatively low. The higher Ct scores from cards may be due to reduced sensitivity of the oral swab samples [8, 20]. It may also be due to the nature of preservation cards: after nucleic acids adhere to their substrate, reduced recovery of viral RNA occurs when eluting and testing [23]. In a study evaluating the detection of a related virus (Dengue: Flaviviridae) from FTA™ cards, RNA recovery was lower from FTA™ cards compared to other preservation methods although the cards demonstrated high sensitivity [23].

The FTA™ and RNASound™ cards were more reliable for American Crows than for any other species, and the sample size for crows was highest. From our 2012 and 2013 trials, FTA™ cards yielded Ct scores for WNV-positive American Crows an average of 4.0 to 5.5 Ct higher than scores from kidney tissue, and this was the smallest difference among the corvids we assessed. American Crows have been shown to have high viremias when infected with WNV and frequently have blood in the oral cavity [24] which may help explain the closer alignment of Ct scores from American Crows among the test types. This can also decrease WNV detection errors in the less sensitive (or specific) nucleic acid preservation cards.

In contrast, in both Trials I and II, Western Scrub-jay and Yellow-billed Magpie results from the FTA™ and/or RNASound™ cards were less accurate than for American Crows. Western Scrub-jay Ct scores from FTA™ cards were an average of 8.0 and 8.1 Ct higher than scores from kidney tissue in Trials I and II, respectively (although in Trial II, results were not significant). An earlier study found that oral swab scores from American Crows agreed with corresponding kidney scores while discrepancies were found in the same dual test comparison on Western Scrub-jays; this may be due to more WNV present in the kidneys than oral cavities of Western Scrub-jays [8].

While discrepancies among test types were expected, the differences unexpectedly occurred in both directions (this was especially prominent in the notable discrepancies). In the WNV- positive crows, there was a significant difference between not just the preservation card results and other test results, but also between kidney tissue, brain tissue, and oral swab results, suggesting different levels of virus among dead birds’ tissues. In an experimental study, corvids infected with WNV showed different amounts of WNV in blood, tracheal and oral swabs, and various organs [25]. In another study, some American Robins infected experimentally with WNV had low levels of WNV RNA in their oral cavity after their blood had cleared infection [26]. Evidently variation can exist in virus titers depending on the time the samples were collected after infection and the tissue type of the infected bird being tested, and this may be one of the contributing factors in our test result discrepancies on wild birds where days post infection were not known.

Most non-corvid passerines and raptors in our study were negative for WNV, but RNASound™ cards detected WNV in three non-corvid passerines, suggesting oropharyngeal samples preserved on the cards from other species may test positive for WNV. Overall sensitivity and specificity of the FTA™ and RNASound™ cards were higher than for RAMP™ or VecTOR™ [8] and are therefore more reliable than these rapid-antigen tests.

## Implementation and Conclusion

In September 2013, a reduction in resources prompted changes to the DBSP, including discontinuation of dead bird necropsy and kidney testing and the implementation of an alternative sampling method relying on oral swabs. In our comparative trials, both filter paper products displayed similar accuracy in detecting WNV RNA from dead bird oral swab samples preserved on the cards. RNASound™ cards were chosen for sample collection because their pre-punched discs expedited laboratory processing with less risk of cross-contamination.

Although dead birds tested with RNASound™ cards in the DBSP in late 2013 and 2014 were not compared to another testing method, we were encouraged that many species of dead birds tested positive besides corvids (which are known to develop extremely high viremia). In 2014, some agencies conducted rapid antigen or RT-PCR testing of dead birds and then took an oral sample onto an RNASound card™ to confirm their results. Thus, we were able to compare 19 dead birds tested via RNASound™ cards to RAMP™ (eight birds), VecTOR™, (seven birds), or RT-PCR analysis of tissue (four birds; brain, kidney, or oral swabs in lysis buffer). RNASound™ card results agreed with tissue RT-PCR results in 16/19 birds (84%). Non-agreement occurred between three RNASound™ cards and rapid-antigen tests (one RAMP™ and two VecTOR™). These results from the 2014 testing season, combined with 2012 and 2013 trial results, added assurance that this sampling and testing method is a suitable substitute for the program’s longstanding kidney tissue testing.

Feedback regarding the new sampling and testing method from participating local agencies has been favorable: carcasses can be sampled immediately and do not need to be maintained at cold temperatures, packaged, and shipped, and agencies may send the cards to the laboratory at their convenience. Agencies receive automatic emails with results by the DART laboratory, allowing them to rapidly respond with enhanced surveillance (i.e., mosquito traps or testing additional birds) and public messaging in the localized area where a WNV-positive dead bird was found. As a cost-effective, time efficient, and accurate alternative for WNV dead bird testing, this protocol using nucleic acid preservation cards to sample dead birds is now fully implemented as part of our WNV Dead Bird Surveillance Program.
